# Inhibition of Autophagy Potentiated the Antitumor Effect of Nedaplatin in Cisplatin-Resistant Nasopharyngeal Carcinoma Cells

**DOI:** 10.1371/journal.pone.0135236

**Published:** 2015-08-19

**Authors:** Zhongyu Liu, Jun Liu, Li Li, Dan Nie, Qilei Tao, Jian Wu, Jiajun Fan, Chen Lin, Shuwei Zhao, Dianwen Ju

**Affiliations:** 1 Department of Otolaryngology-Head and Neck Surgery, Changzheng Hospital, Second Military Medical University, Shanghai, China; 2 Mary Babb Randolph Cancer Center, West Virginia University, Morgantown, West Virginia, United States of America; 3 Department of Otolaryngology, Traditional Chinese Medicine Hospital of Guangdong Province, Guangzhou, China; 4 Department of Biosynthesis, School of Pharmacy, Fudan University, Shanghai, China; Central South University, CHINA

## Abstract

Nedaplatin, a cisplatin analog, was developed to reduce the toxicity of cisplatin, whereas it can be cross-resistant with cisplatin in some circumstances. This study aimed to investigate the role of autophagy in nedaplatin induced cell death in cisplatin-resistant nasopharyngeal carcinoma cells. Here, we showed that HNE1/DDP and CNE2/DDP cells were resistant to nedaplatin-induced cell death with reduced apoptotic activity. Nedaplatin treatment resulted in autophagosome accumulation and increased expression of LC3-II, indicating the induction of autophagy by nedaplatin in HNE1/DDP and CNE2/DDP cells. Inhibition of autophagy by Bafilomycin A1 (Baf A1) and 3-Methyladenine (3-MA) remarkably enhanced the antitumor efficacy of nedaplatin in HNE1/DDP and CNE2/DDP cells, suggesting that the resistance to nedaplatin-induced cell death was caused by enhanced autophagy in nedaplatin-resistant NPC cells. Additionally, Baf A1 enhanced reactive oxygen species (ROS) generation and apoptosis induced by nedaplatin in HNE1/DDP cells. Mechanistically, nedaplatin treatment caused activation of ERK1/2 and suppression of Akt/mTOR signaling pathways. While inhibition of ERK1/2 by MEK1/2 inhibitor, U0126, could reduce the expression of LC3-II in nedaplatin-resistant NPC cells. Furthermore, suppression of ROS could inhibit nedaplatin-induced ERK activation in HNE1/DDP cells, indicating that ROS and ERK were involved in nedaplatin-induced autophagy. Together, these findings suggested that autophagy played a cytoprotective role in nedaplatin-induced cytotoxicity of HNE1/DDP and CNE2/DDP cells. Furthermore, our results highlighted a potential approach to restore the sensitivity of cisplatin-resistant nasopharyngeal cancer cells to nedaplatin in combination with autophagy inhibitors.

## Introduction

Nasopharyngeal carcinoma (NPC) is a type of cancer arising from the epithelial cells that line the nasopharynx. NPC is considered to be a rare cancer globally, whereas it is endemic in the southeastern Asia, particularly in Southern China [1]. The current standard treatment for patients with stage I nasopharyngeal cancer is radiotherapy (RT) alone, and those with stage II-IVB disease are treated with concurrent chemoradiotherapy [2]. Although cisplatin-based chemotherapy is the first-line treatment for locoregionally advanced nasopharyngeal carcinoma [3,4], the clinical application of cisplatin has been limited due to its toxicity and acquired resistance developed during the therapy. Nedaplatin is the second generation of platinum complex, which was developed to reduce toxicities, such as nephrotoxicity and gastrointestinal toxicity, commonly seen in cisplatin-treated patients [5]. Nedaplatin-based chemotherapy is an effective and safe treatment for patients with locoregionally advanced nasopharyngeal carcinoma [6–8]. However, it has been documented that nedaplatin was cross-resistant with cisplatin in the L1210/CDDP leukemia mode [9]. Acquired resistance to antitumor drugs is a major cause of cancer relapse and cancer-related mortality. Therefore, approaches to enhance the sensitivity of NPC to chemotherapies have generated a great deal of interests.

Autophagy is a highly conserved cellular process by which cytoplasmic components are sequestered in autophagosomes and delivered to lysosomes for degradation [10]. Autophagy is essential for survival, differentiation, development, and homeostasis in eukaryotic cells. Dysregulation of autophagy contributes to a number of diseases, including cancer [11]. However, the role of autophagy in cancer is characterized by double-edged sword. Autophagy can promote tumor suppression during cancer initiation. Conversely, it can be tumor-promoting in established cancers [12]. Since autophagy is substantially activated in cancer cells, it may involve in drug resistance by facilitating cancer cell survival during metabolic stresses caused by anticancer agents [13]. For instance, upregulation of autophagy resulted in resensitization of H460/cis cells (cisplatin-resistant lung cancer cells) to cisplatin-induced cell death [14]. However, conflicting evidence showed that inhibition of autophagy resensitized SKOV3/DDP cells (cisplatin-resistant ovarian cancer cells) to cisplatin [15]. Moreover, the relationship between autophagy and drug-resistance is intricate since there exist some common regulatory elements, including ROS [16,17], PI3K/Akt/mTOR pathway and ERK pathway [18].

Nevertheless, it is still unclear whether autophagy is involved in nedaplatin-induced cell death in cisplatin-resistant NPC cells.

In this study, we presented evidences demonstrating that nedaplatin was cross-resistant with cisplatin. Meanwhile, autophagy was induced in HNE1/DDP cells and CNE2/DDP after they were exposed to nedaplatin. Suppression of autophagy significantly enhanced apoptosis, ROS generation and growth inhibition induced by nedaplatin. Moreover, the Akt/mTOR and ERK1/2 signaling pathways were involved in autophagy induced by nedaplatin. Taken together, our results revealed that targeting autophagy promoted the antitumor effect of nedaplatin in cisplatin-resistant NPC cells. Our results may lead to the development of nedaplatin in combination with autophagy inhibitors as a potential therapy regime for NPC with cross-resistance of nedaplatin and cisplatin.

## Materials and Methods

### Regents and Chemicals

Cisplatin was purchased from Gejiu Bio-pharmaceutical Co., Ltd (Yunnan, China). Nedaplatin was purchased from Aosaikang Pharmaceutical Co., Ltd (Jiangsu, China), Baf A1 and 3-MA were purchased from Sigma (St Louis, MO, USA). Cyto-ID Autophagy Detection Kit was purchased from Enzo Life Sciences, Inc (Farmingdale, NY, USA). Apoptosis Detection Kit was purchased from BD Biosciences (Franklin Lakes, NJ, USA). Obtained from Beyotime Institute of Biotechnology (Haimen, Jiangsu Province, China) were 2′,7′-dichlorodihydrofluorescein diacetate (DCFH-DA) and N-acetyl-L-cysteine (NAC).Antibodies of human LC3, Beta-actin, The MEK1/2 inhibitor U0126,Phospho-mTOR (Ser2448), Cleaved Caspase-3,Phospho-p44/42 MAPK(Erk1/2)(Thr202/Tyr204) and p44/42 MAPK(Erk1/2) were purchased from Cell Signaling Technology (Danvers, MA, USA). Obtained from Epitomics (Burlingame, CA, USA) were the Anti-p70 S6 Kinase Phospho (pS371) and PARP-1 Phospho(p116/p85). Obtained from MR Biotech (Shanghai, China) were the secondary antibodies horseradish peroxidase (HRP)-conjugated goat anti-mouse and anti-rabbit immunoglobulin G. All the other antibodies were obtained from Sigma (St Louis, MO, USA).

### Cell culture

Cisplatin-sensitive nasopharyngeal cancer cell lines CNE2, HNE1 and its cisplatin-resistant clone HNE1/DDP were purchased from the Central Laboratory of Xiangya School of Medicine, Central South University. CNE2/DDP cell lines were kindly provided by the Department of Hematology, Zhujiang Hospital, Southern Medical University. Cells were cultured in RPMI-1640 medium (Invitrogen, San Diego, CA, USA) supplemented with 10% heat-inactivated fetal bovine serum (Invitrogen, San Diego, CA, USA), 2 mM L-glutamine, 100 U/mL penicillin, 100 μg/mL streptomycin in a humidified incubator at 37°C and 5% CO_2_. Besides, cisplatin-resistant HNE1/DDP and CNE2/DDP cells were maintained in RPMI-1640 10% fetal bovine serum medium medium containing 1 μg/mL cisplatin to maintain resistance.

### Cell viability assay

Cellular viability was measured by MTT cytotoxicity assay. Briefly, cells were plated at 6×10^3^ (HNE1 and HNE1/DDP) and 1×10^4^ (CNE2 and CNE2/DDP) per well and cultured for 24 h. After that, cells were treated with various concentrations of cisplatin or nedaplatin with or without autophagy inhibitors for 48 h. Cells were incubated with MTT (0.5 mg/mL) for 4 h at 37°C. After the supernatant was carefully removed, 100 μL of dimethylsulphoxide was added and the absorbance of the solution was measured at a wavelength of 570 nm.

### Apoptosis assay

Cell apoptosis was detected by Annexin VFITC/PI Detection Kit (BD Biosciences, San Diego, CA, USA) according to manufacturer’s instructions. After exposure to different experimental conditions for 48 h, HNE1/DDP cells were harvested, washed twice with cold PBS, and resuspended in binding buffer at a concentration of 1×10^6^ cells/mL. Then, the cells were incubated with annexin V-FITC and PI for 15 min in the dark according to the manufacturer's protocol. Afterwards, samples were analyzed by flow cytometry using Calibur flow cytometer (Becton-Dickinson, Fullerton, CA, USA).

### Transmission electron microscopy

After designated treatment, HNE1/DDP cells were fixed with 2% glutaraldehyde in 0.1 M PBS (pH 7.3) for 2 h at 4°C and washed extensively with 0.1 M cacodylate buffer including 0.1% CaCl_2_. Samples were fixed in 0.1 M cacodylate buffer containing 0.1% CaCl_2_ for 30 min and then dehydrated through a graded series of ethanol and polymerized at 60°C for 48h. After being cut by ultracut microtome, the sections were stained with uranyl acetate and lead citrate. Subsequently, a JEM 1230 transmission electron microscope (JEOL, USA) was used to examine the section samples at a voltage of 60 kV.

### Western blot analysis

After treatment with indicated condition, cells were harvested and washed with cold phosphate-buffered saline (PBS) and then incubated in Cell Lysis Buffer (Beyotime Biotchnology, China) in ice for at least 20 min. The lysates were centrifuged at 12,000×g for 10 min and the supernatants were collected. Protein concentrations were measured by the bicinchoninic acid (BCA) method. Equivalent amount of protein was run on SDS-PAGE gels and subsequently electro-transferred to polyvinylidene fluoride (PVDF) membranes. Membranes were blocked with 5% BSA for 1 h at room temperature, and then incubated overnight at 4°C with primary antibodies, followed by incubation with secondary antibodies at room temperature for 2 h. the protein signals were developed using an enhanced chemiluminescent detection kit (Pierce, Rockford, IL, USA).

### Confocal Microscopy

Cells were seeded in cell culture dishes with glass bottoms and allowed to recover overnight. After treatment with 6 μg/ml nedaplatin for 0 h and 24 h, the cells were disposed with Cyto-ID Autophagy Detection Kit according to the manufacturer’s protocol. Briefly, after being stained with Cyto-ID Green dye and Hoechst 33342 for 30 min, cells were washed and re-suspended with 1x Assay Buffer and immediately analyzed with an Olympus fluorescence microscope. As a positive control, cells were treated with 500 nM of Rapamycin for 4 h and disposed with the same procedures.

### Measurement of intracellular ROS

Intracellular ROS was determined by a fluorometric assay (DCF-DA assay). HNE1/DDP cells were seeded in 96-well microplates at the density of 6×10^3^ cells/well and grown for 24 h. After cells were treated with nedaplatin with or without NAC, U0126 and Baf A1 for the indicated time periods, HNE1/DDP cells were loaded with 10 μM DCFH-DA for 20 min at 37°C, and the generation of ROS was determined using Tecan M1000 Multi-Mode Microplate Reader (TECAN, Switzerland) with the excitation and emission wavelengths set at 488 and 525 nm. Relative DCF fluorescence intensity of treated cells was expressed as a percentage of control (as 100%).

### Statistics analysis

Data were expressed as means ± S.D. Statistics analysis was carried out with GraphPad Prism 5. Comparisons between two treatment groups were performed using Student’s ***t*** test (two-tailed) and One-way Anova, and *p* < 0.05 was considered to be statistically significant.

## Results

### HNE1/DDP and CNE2/DDP cells show resistance to nedaplatin-induced cell death

**Fig 1:**
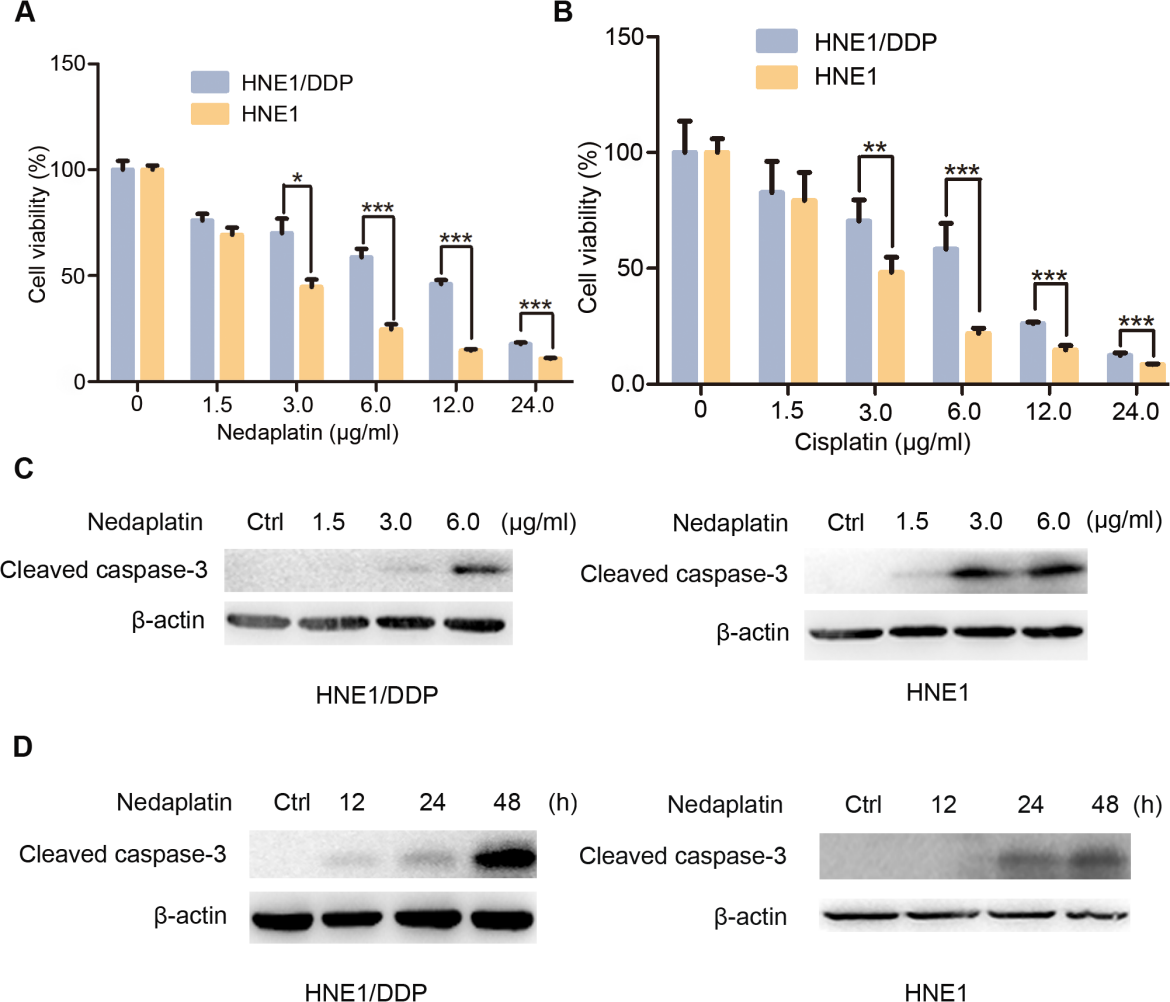
HNE1/DDP cells were resistant to nedaplatin-induced cell death. (A&B) HNE1 cells and HNE1/DDP cells were treated with the indicated concentrations of nedaplatin (A) or cispaltin (B) for 48 h. Cell viability was determined by MTT assay at the wavelength of 570 nm. Data are mean ± SD from five independent experiments. *p<0.05, **p<0.01 and ***p<0.0001 compared to HNE1 cells. (C) Western blot analysis for the expression of cleaved caspase 3 protein in HNE1 and HNE1/DDP cells treated with the indicated concentrations of nedaplatin for 48 h. (D) Western blot analysis for the expression of cleaved caspase 3 protein in HNE1 and HNE1/DDP cells treated with 6.0 μg/ml nedaplatin as indicated time.

To assess whether cisplatin-resistant NPC cells would resist to nedaplatin-induced cell death, both cisplatin-sensitive nasopharyngeal cancer cells and cisplatin-resistant NPC cells were treated with nedaplatin at increasing doses for 48 h. In a parallel set of experiment, the cells (HNE1, CNE2, HNE1/DDP and CNE2/DDP) were treated with cisplatin for comparison. As shown in Fig 1A and 1B and S1 Fig, both nedaplatin and cisplatin induced dose-dependent cell death of HNE1 and CNE2 cells with IC50 (2.75±0.37 μg/ml for nedaplatin vs. 2.83±0.25 μg/ml for cisplatin, 1.49±0.20 μg/ml for nedaplatin v.s. 1.24±0.15 μg/ml for cisplatin, respectively). In contrast, the cisplatin-resistant HNE1/DDP and CNE2/DDP cells were less sensitive to nedaplatin-induced cell death with the IC50 of 6.74±0.58 μg/ml and 7.86±0.73 μg/ml, which was similar to its IC50 for cisplatin (6.02±0.72 μg/ml and 6.21±0.58 μg/ml, respectively). These results were in line with previous observation, indicating that nedaplatin was cross-resistant with cisplatin [19]. Since previous study showed that nedaplatin induced tumor cell death through induction of apoptosis [20], we reasoned that the resistance to nedaplatin was most likely due to a lack of apoptosis [21]. To test our hypothesis, we treated HNE1 and HNE1/DDP cells with nedaplatin and analyzed the level of cleaved form of caspase 3, a typical marker for apoptotic cell death. As shown in Fig 1C, nedaplatin treatment significantly promoted the cleavage of caspase 3 in HNE1 cells, while no obvious cleavage caspase 3 could be detected in nedaplatin-treated HNE1/DDP cells. Moreover, time-course experiment showed that nedaplatin-induced cleavage of caspase 3 in HNE1/DDP cells was delayed by 24 hours compared to HNE1 cells. (Fig 1D). These results indicated that cisplatin-resistant NPC cells (HNE1/DDP and CNE2/DDP cells) were resistant to nedaplatin.

### Induction of autophagy by nedaplatin in NPC cells

**Fig 2:**
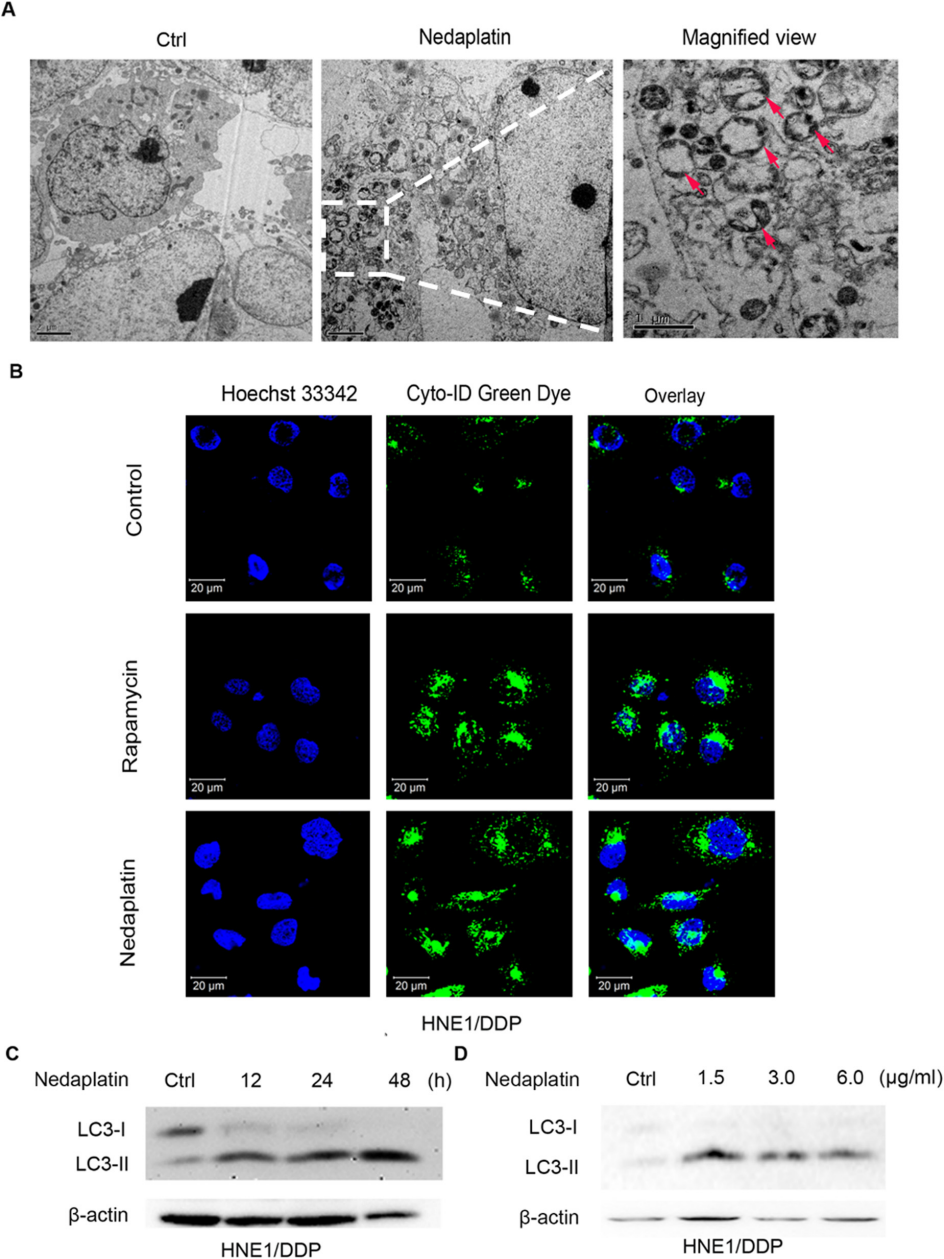
Autophagy was induced by nedaplatin in HNE1/DDP cells. (A) Nedaplatin induced formation of autophagosomes. HNE1/DDP cells were either untreated or treated with 6.0 μg/ml of nedaplatin for 48 h. A magnified view of the electron photomicrograph shows a characteristic autophagosome. (B) HNE1/DDP cells were treated with 6.0 μg /ml of nedaplatin for 24 h or with 500 nM of rapamycin for 4 h, and then the cells were stained with Cyto-ID Green autophagy dye and analyzed by confocal microscopy. (C) Immunoblot analysis of LC3-I/II levels. HNE1/DDP cells were treated with 6.0 μg/ml nedaplatin for 12, 24, and 48 h. (D) Immunoblot analysis of LC3-I/II levels. HNE1/DDP cells were treated with nedaplatin for 48 h at 0, 1.5, 3.0 and 6.0 μg/ml.

Autophagy occurs at low basal levels in all cells and is rapidly up-regulated when cell faces stress. The ability of autophagy to promote cell survival during metabolic stress may promote resistance to cytotoxic therapy [22]. To assess whether nedaplatin induced autophagy in NPC cells, we initially examined the accumulation of autophagosomes by transmission electron microscopy (TEM). As shown in Fig 2A, nedaplatin-treated HNE1/DDP cells showed numerous cytosolic autophagic vacuoles with clearly double-layered membrane structures compared with untreated cells. Since autophagosome was recognized as characteristic double membrane vacuolar structures containing cytoplasmic contents [23], our results indicated that nedaplatin might induce autophagosome accumulation in HNE1/DDP cells. To confirm the TEM result, nedaplatin-treated HNE1/DDP cells were incubated with autophagic vacuoles specific marker, Cyto-ID, for 30 min and fixed for confocal fluorescence microscopy analysis. As shown in Fig 2B, treatment of HNE1/DDP cells either with nedaplatin or autophagy inducer, rapamycin, resulted in a substantial increase in autophagosomes in the cells. Similar results were discovered in nedaplatin-treated HNE1 and CNE2/DDP cells (S2E and S2F Fig).

Microtubule-associated protein light chain 3 (LC3) is a well-established protein marker for monitoring autophagy process [24]. Increased LC3-II level and LC3-II/LC3-I ratio indicate the occurrence of autophagy [17]. Consistent with the results of morphological studies, nedaplatin induced time- and dose-dependent increase in LC3-II levels in HNE1, HNE1/DDP and CNE2/DDP cells (Fig 2C and 2D, S2A–S2D Fig).

Taken together, these results suggested that autophagy was activated by nedaplatin in NPC cells.

### Inhibition of autophagy enhances cytotoxic effect of nedaplatin in cisplatin-resistant NPC cells

**Fig 3:**
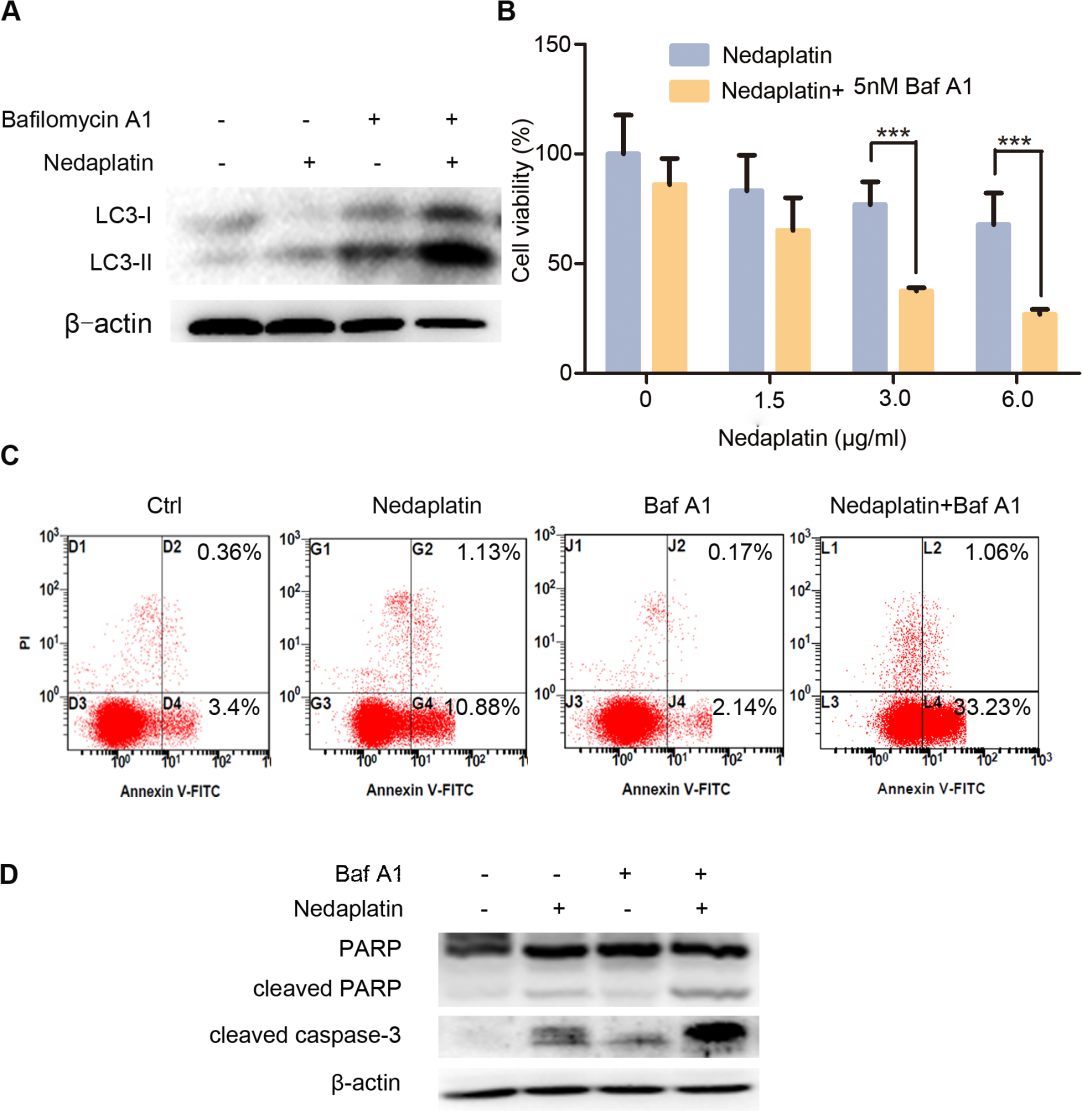
Inhibition of autophagy enhanced nedaplatin-induced apoptosis and suppression of cell growth in HNE1/DDP cells. (A) HNE1/DDP cells were incubated with 6.0 μg/ml nedaplatin for 48 h, in the presence or absence of Baf A1 (5 nM) for 48 h, and the levels of LC3-I/II were detected by western blot. (B)HNE1/DDP cells were untreated or treated with nedaplatin at indicated concentrations in the absence or presence of Baf A1 (5 nM) for 48h. The cell viability was determined by MTT assay at the wavelength of 570 nm. Data are mean ± SD from five independent experiments. ***p<0.001 compared to nedaplatin only. (C) HNE1/DDP cells were treated with 6.0 μg/ml with or without Baf A1 (5 nM) for 48 h and then analyzed by FACS after PI and FITC-annexin V staining. (D) HNE1/DDP cells were incubated with or without 6.0 μg/ml of nedaplatin in the presence or absence of the autophagy inhibitors Baf A1 for 48 h. The whole protein was extracted, and PARP, cleaved PARP and cleaved caspase 3 were analyzed by Western blot.

Given that nedaplatin-treated HNE1/DDP and CNE2/DDP cells exhibited significant autophagic activity, we further tested whether enhanced autophagy was associated with the cell death resistance in response to nedaplatin. Baf A1 inhibits autophagy by blocking the fusion of autophagosome and lysosome and leads to significant elevation of LC3-II expression. Western blot analysis revealed that the addition of Baf A1 to nedaplatin significantly enhanced LC3-II expression in HNE1/DDP cells when compared to the cells treated with nedaplatin alone (Fig 3A), while treatment with Baf A1 alone did not affect the growth of HN1/DDP cells (data not shown). Interestingly, inhibition of autophagy by Baf A1 substantially potentiated nedaplatin-induced cell death (Fig 3B). Similar results were obtained in CNE2/DDP cells (S3D and S3E Fig). To understand the role of autophagy in nedaplatin-induced apoptosis, apoptotic rate of HN1/DDP cells was determined after they were co-incubated with nedaplatin and Baf A1. Compared with the cells treated with nedaplatin alone, HNE1/DDP cells treated with both nedaplatin and Baf A1 exhibited significant increases in apoptotic rate (Fig 3C) as well as the protein levels of cleaved caspase 3 and cleaved PARP (Fig 3D). Moreover, another autophagy inhibitor 3-MA was used to confirm these results, which could block autophagy initiation and lead to the decrease in LC3-II level. As illustrated in S3A–S3C Fig, inhibition of autophagy using 3-MA significantly enhanced nedaplatin-induced cell death and caspase 3 cleavage in HNE1/DDP cells.

Taken together, these results suggested that inhibition of autophagy could enhance nedaplatin-induced apoptotic activity and cell death of cisplatin-resistant NPC cells.

### ROS plays an essential role in nedaplatin-induced autophagy and cell growth inhibition

**Fig 4:**
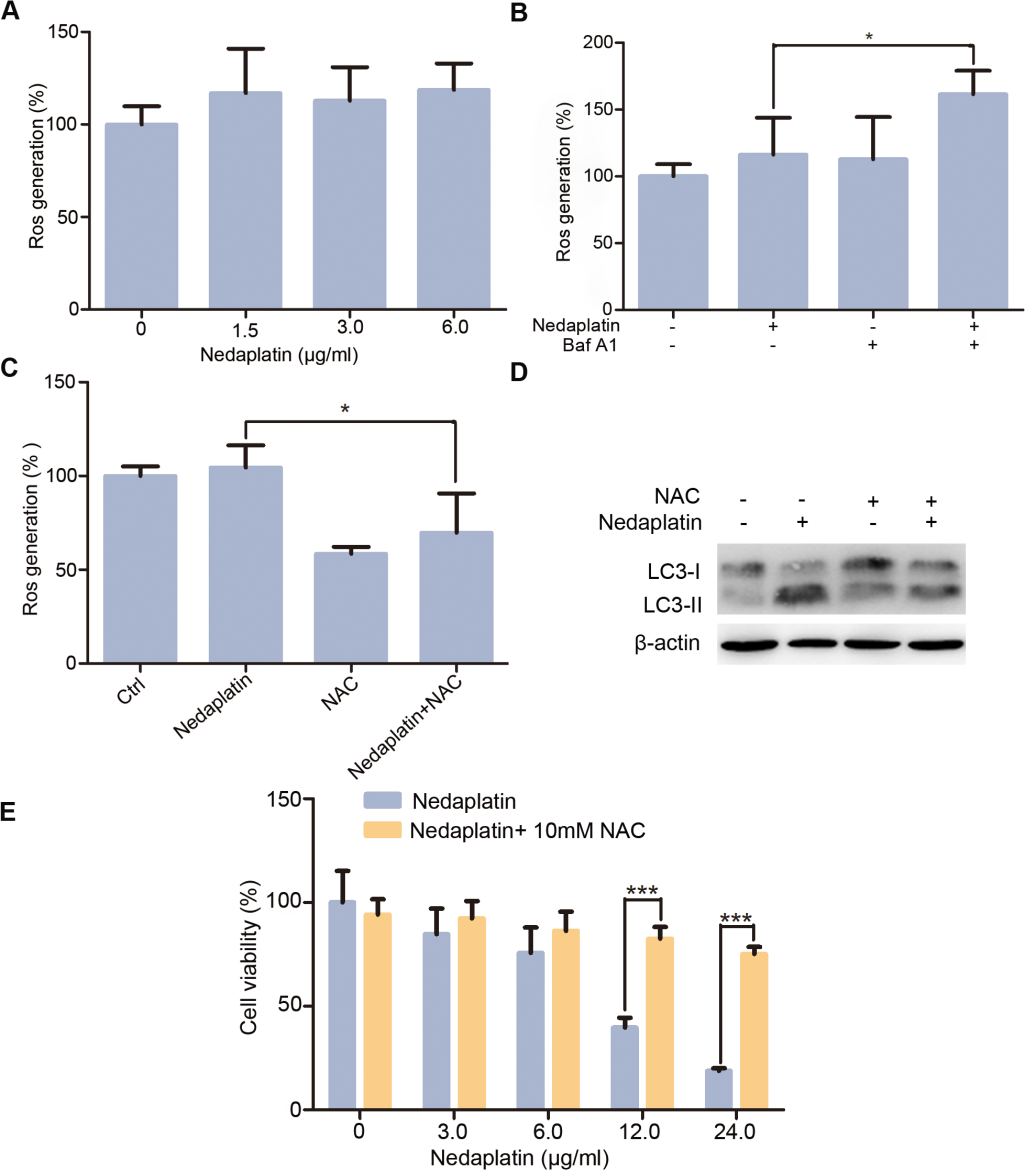
Suppression of ROS production inhibited nedaplatin-induced cell death. (A) HNE1/DDP cells were incubated with varied concentrations of nedaplatin for 12 h. Then, the samples were prepared as described in Materials and Methods. All data are expressed as means ± SD from five independent experiments. *p< 0.05. (B) HNE1/DDP cells were incubated with 6.0 μg/ml nedaplatin in the presence or absence of Baf A1 for 6 h. Then, the cells were treated as described in A. (C) HNE1/DDP cells were treated with 6.0 μg/ml nedaplatin for 12 h in the absence or presence of NAC (10 mM). The cells were treated as described in A. *p<0.05. (D) The LC3 I/II levels were examined by western blot after the nedaplatin treatment with or without of NAC (10 mM) for 48 h. (E) HNE1/DDP cells were treated with 6.0 μg/ml nedaplatin for 48 h in the absence or presence of NAC (10 mM). The cell viability was determined as described in Materials and Methods. Data are mean ± SD from five independent experiments. ***p<0.001 compared to nedaplatin only.

Previous report has demonstrated that ROS played an important role in cisplatin-induced cell death [25]. To explore whether nedaplatin treatment induced ROS generation in HNE1/DDP cells, we applied DCFH-DA, a well-established compound to detect and quantify intracellular generated ROS. Our results showed that treatment of nedaplatin for 12 h did not lead to obvious change of ROS level in HNE1/DDP cells (Fig 4A). The lack of ROS production in response to nedaplatin stimulation may be caused by up-regulation of autophagy in HNE1/DDP cells, since there was evidence to demonstrate that ROS accumulation was suppressed by autophagy [26]. To test this idea, HNE1/DDP cells were treated with autophagy inhibitor, BafA1, and the level of ROS production in response to nedaplatin stimulation was measured. As shown in Fig 4B, in the presence of autophagy inhibitor, BafA1, nedaplatin resulted in 2.78-fold ROS production in HNE1/DDP cells over nedaplatin alone treated cells. To further verify that ROS contributed to nedaplatin-induced cell killing effect, HNE1/DDP cells were pretreated with an antioxidant, NAC. In the presence of NAC, ROS level in HNE1/DDP cells was significantly reduced (Fig 4C). Previous studies showed that autophagy was induced under oxidative stress [27,28]. Consistently, we found that pretreatment with NAC remarkably reduced LC3-II conversion (Fig 4D), indicating that NAC could block nedaplatin-induced autophagy. Further, the combination of nedaplatin with NAC could rescue HNE1/DDP cells from nedaplatin-induced cell death (Fig 4E). These results indicated that accumulation of ROS played a critical role in the resensitization of HNE1/DDP cells to nedaplatin-induced cell death under conditions of suppressed autophagy.

### Involvement of PI3K/Akt/mTOR and ERK1/2 signaling pathways in nedaplatin-induced autophagy

**Fig 5:**
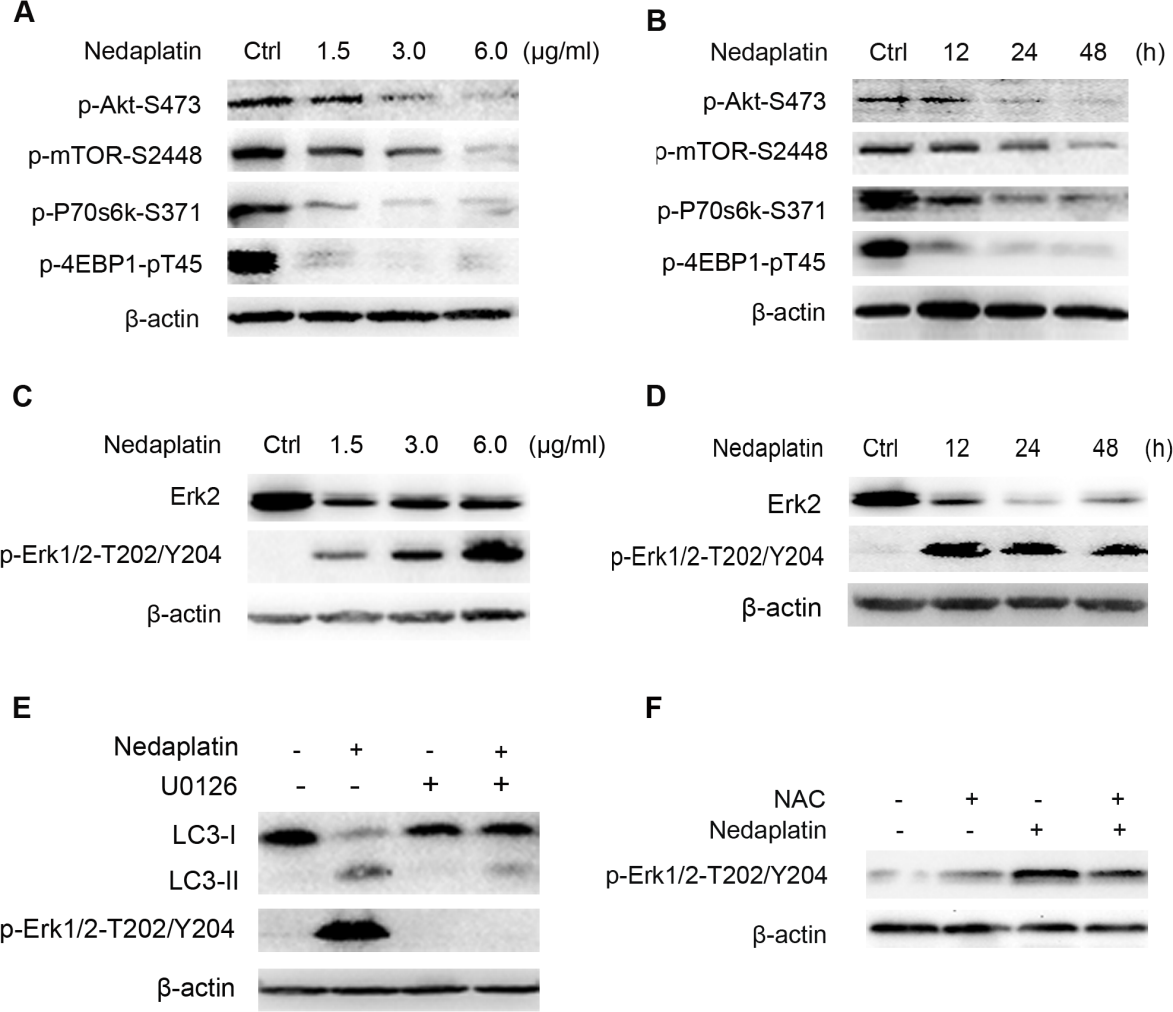
Akt/mTOR and ERK signaling pathways were involved in nedaplatin-induced autophagy in HNE1/DDP cells. (A) HNE1/DDP cells were treated with different concentrations of nedaplatin for 48 h. Levels of pAkt, pmTOR, pP70S6K, and p4E-BP1 were detected by western blot. (B) HNE1/DDP cells were treated with 6.0 μg/ml nedaplatin as indicated times. Levels of pAkt, pmTOR, pP70S6K, and p4E-BP1 were detected by western blot. (C) HNE1/DDP cells were treated with different concentrations of nedaplatin for 48 h. Protein extracts were analyzed using Erk1/2 and phospho-Erk1/2 (Thr202/Tyr204) by western blot. (D) HNE1/DDP cells were treated with 6.0 μg/ml nedaplatin as indicated times. Protein extracts were analyzed using Erk1/2 and phospho-Erk1/2(Thr202/Tyr204) by western blot. (E) HNE1/DDP cells were treated with 6.0 μg/ml nedaplatin for 48 h with or without the pretreatment of U0126 (20 μM) for 2h. Protein extracts were examined by Western blot using LC3 I/II and phospho-Erk1/2 (Thr202/Tyr204) antibodies. (F) The phospho-Erk 1/2 (Thr202/Tyr204) levels were examined by western blot after the nedaplatin treatment with or without of NAC (10 mM) for 48 h.

Our result demonstrated that inhibition of mTOR by rapamycin induced autophagy in HNE1/DDP cells (Fig 2B), suggesting that PI3K/Akt/mTOR may be involved in nedaplatin-induced autophagy in HNE1/DDP cells. To test this speculation, HNE1/DDP cells were treated with nedaplatin, and the phosphorylation of key components of PI3K/Akt/mTOR pathway including Akt, mTOR, p70S6K and, 4E-BP1 were examined by western blotting analysis. As shown in Fig 5A and 5B, nedaplatin treatment caused a time- and does-dependent inhibition of phosphorylation of AKT, mTOR, p70S6K and 4E-BP1, which was consistent with previous reports showing that autophagy was suppressed by activated PI3K/Akt/mTOR signaling pathway [29,30]. Additionally, Sustained ERK activity that occured in tumors has been shown previously to have prosurvival effect by contributing to autophagy [31]. To test whether ERK was involved in nedaplatin-induced autophagy, HNE1/DDP cells were treated with nedaplatin, and phosphorylation of ERK was examined by Western blot analysis. As illustrated in Fig 5C and 5D, nedaplatin induced a time- and does-dependent increase in phospho-ERK1/2 in HNE1/DDP cells. Phosphorylation of ERK1/2 occured 12 hours after nedaplatin treatment and sustained until 48 hours after the treatment. Inhibition of ERK1/2 phosphorylation by MEK1/2 inhibitor, U0126, blocked nedaplatin-induced increase in LC3-II level (Fig 5E), indicating that nedaplatin-induced autophagy was mediated by MEK/ERK1/2 signaling pathway.

In addition, inhibiting ERK could rescue Akt and mTOR inhibition induced by nedaplatin, suggesting potential relationship between ERK and Akt/mTOR signaling pathways in HNE1/DDP cells (S4A Fig). It was reported that ROS could regulate AMPK/ERK signaling pathway [32,33]. The current study indicated that inhibiting ERK could not significantly change ROS generation, while inhibiting ROS generation could obviously inhibit nedaplatin-induced ERK activation in HNE1/DDP cells (Fig 5F and S4B Fig). Consistent with the previous finding [34], our data suggested that ROS generation might be the upstream of ERK pathway in HNE1/DDP cells.

Taken together, these results suggested that suppression of Akt/mTOR and activation of ERK1/2 signaling pathways were involved in nedaplatin-induced autophagy in HNE1/DDP cells.

## Discussion

Cisplatin-based chemotherapy has showed potent anti-NPC efficacy, providing significant survival benefit for patients over the decades. However, the acquired resistance and serve toxicity of cisplatin limited its application in clinic. Nedaplatin, a second-generation of cisplatin analog, has similar anticancer potencies with significantly fewer side effects, especially less nephrotoxicity and gastrointestinal reaction [35,36]. It has been used as a substitute for cisplatin for the treatment of locoregionally advanced NPC. However, due to cross-resistance with cisplatin, nedaplatin is limited for clinical application and efficacy. In this study, we investigated the potential role of autophagy in nedaplatin-induced cell death in cisplatin-resistant HNE1/DDP cell line. Autophagy is functional at low levels in normal tissues, but is upregulated in settings of metabolic stress, such as nutrient depletion and the presence of apoptosis activators [37,38]. Recent studies have revealed that cisplatin induced autophagy in cancer cells [39] and the up-regulation of autophagy in multi-drug resistance cells allowed cancer cells to survive during chemotherapy stress [40]. In this study, Our current study showed that HNE1/DDP cells and CNE2/DDP were resistant to nedaplatin-induced cell death. Furthermore, several standard experiments for detecting autophagy, including transmission electron microscopy, confocal microscopy and Western blot analysis, were conducted to demonstrate that autophagy was induced in nedaplatin-treated HNE1/DDP and CNE2/DDP cells. In fact, autophagy is a double-edged sword in cancer therapy. In some cases, autophagy served as a protective mechanism to mediate drug resistance phenotype during anti-cancer therapy. For instance, in drug-resistant esophageal squamous carcinoma cells, suppression of autophagy markedly enhanced 5-FU-induced cell killing [41]. However, co-treatment of cisplatin with trifluorperazie, a well-known autophagic inducer, overcomed the resistance of the H460/cis cells [13], suggesting the cytotoxic role of autophagy. In this study, we found that Baf A1 and 3-MA inhibited autophagy and greatly sensitized HNE1/DDP and CNE2/DDP cells to nedaplatin-induced cell death through the enhancement of apoptosis. These results were consistent with previous report [41] and thus suggested that autophagy was an important survival mechanism for HNE1/DDP cells.

Generally, ROS plays an important role in different types of cell death. Recent study showed that the increase in ROS could overcome the cisplatin-resistance in lung cancer cells [42]. Our data demonstrated that treatment with nedaplatin alone slightly enhanced ROS generation in HNE1/DDP cells. Furthermore, we showed that inhibition of autophagy significantly increased the intracellular ROS level in HNE1/DDP cells, which was supported by previous findings showing that inhibition of autophagy resulted in augment of ROS accumulation in other types of cancer cells [43]. Moreover, pretreatment with NAC decreased autophagy and ROS induced by nedaplatin and rescued tumor cells from the cytotoxic effects of nedaplatin, suggesting that accumulation of ROS was an important mechanism in the resensitization of HNE1/DDP cells to nedaplatin.

In addition, we also observed that the levels of phosphorylated Akt, mTOR, p70S6K and 4E-BP1 were significantly decreased in nedaplatin-treated HNE1/DDP cells. Consistent with our findings, previous studies showed that the Akt/mTOR/p70S6K/ pathway was a negative regulator of autophagy [44]. However, the ERK1/2 pathway positively regulated autophagy [45]. In the current study, ERK1/2 activation appeared to contribute to nedaplatin-induced autophagy, because inhibition of MEK/ERK1/2 signaling pathway attenuated nedaplatin–triggered autophagy. Moreover, elimination of ROS by NAC significantly attenuated the activation of ERK, indicating that ROS acted as a early mediator in regulating ERK pathway. Together, our results suggested that nedaplatin induced autophagy through activation of MEK/ERK1/2 and suppression of PI3K/Akt/mTOR signaling pathways concurrently.

Accumulating evidence suggested that autophagy was an important reason for cancer resistance. A recent study reported that higher level of autophagy in melanomas patients showed lower response rate to BRAF inhibitor [46], suggesting closely relationship between autophagy and cancer resistance in clinic. Moreover, high expression of Beclin-1 was reported to be correlated with poor prognosis of NPC patients [47], indicating that autophagy was closely associated with resistance of NPC in clinical. These findings suggested the potential application of modulating autophagy in NPC treatment.

In conclusion, we demonstrated that nedaplatin induced significant autophagy in cisplatin-resistant NPC cells. Importantly, inhibition of autophagy enhanced the sensitivity of cisplatin-resistant NPC cells to nedaplatin. Moreover, autophagy was associated with ROS accumulation and the activation of apoptosis, ERK and Akt/mTOR/p70S6K/4E-BP1 pathway in cisplatin-resistant NPC cells. Our results suggested that autophagy played a crucial role in the nedaplatin-resistance in the HNE1/DDP cells and further highlighted a potential approach to restore the sensitivity of acquired therapy-resistant NPCs to chemotherapies by a combination of nedaplatin with autophagy inhibitors.

## Supporting Information

S1 Fig CNE2/DDP cells was resistance to nedaplatin. (A) CNE2 cells and CNE2/DDP cells were treated with the indicated concentrations of cisplatin for 48 h. The cell viability was determined by MTT assay at the wavelength of 570 nm. (B) CNE2 cells and CNE2/DDP cells were treated with the indicated concentrations of nedaplatin for 48 h. The cell viability was determined by MTT assay at the wavelength of 570 nm.
(TIF)

S2 Fig Autophagy was induced by nedaplatin in HNE1 and CNE2/DDP cells. (A) Immunoblot analysis of LC3-I/II levels. HNE1 cells were treated with 6.0 μg/ml nedaplatin for 12, 24, and 48 h. (B) Immunoblot analysis of LC3-I/II levels. HNE1 cells were treated with nedaplatin for 48 h at 0, 1.5, 3.0 and 6.0 μg/ml. (C) Immunoblot analysis of LC3-I/II levels. CNE2/DDP cells were treated with 6.0 μg/ml nedaplatin for 12, 24, and 48 h. (D) Immunoblot analysis of LC3-I/II levels. CNE2/DDP cells were treated with nedaplatin for 48 h at 0, 1.5, 3.0 and 6.0 μg/ml.(E) HNE1 cells were treated with 6.0 μg /ml of nedaplatin for 24 h or with 500 nM of rapamycin for 4 h, and then the cells were stained with Cyto-ID Green autophagy dye and analyzed by confocal microscopy. (F) CNE2/DDP cells were treated with 6.0 μg /ml of nedaplatin for 24 h or with 500 nM of rapamycin for 4 h, and then the cells were stained with Cyto-ID Green autophagy dye and analyzed by confocal microscopy.
(TIF)

S3 Fig Inhibition of autophagy enhanced nedaplatin-induced growth inhibition in HNE1/DDP and CNE2/DDP cells. (A) HNE1/DDP cells were incubated with 6.0 μg/ml nedaplatin for 48 h, in the presence or absence of 3-MA (1.5 mM) for 48 h, and the levels of LC3-I/II were detected by western blot. (B) HNE1/DDP cells were untreated or treated with nedaplatin at indicated concentrations in the absence or presence of 3-MA (1.5 mM) for 48h. The cell viability was determined by MTT assay at the wavelength of 570 nm (n = 5, means±SD, ***p<0.001 vs. each respective nedaplatin group). (C) HNE1/DDP cells were incubated with or without 6.0 μg/ml of nedaplatin in the presence or absence of the autophagy inhibitors 3-MA (1.5 mM) for 48 h. The whole protein was extracted, and PARP, cleaved PARP and cleaved caspase-3 were analyzed by western blot. (D) CNE2/DDP cells were incubated with 6.0 μg/ml nedaplatin for 48 h, in the presence or absence of Baf A1 (10 nM) for 48 h, and the levels of LC3-I/II were detected by western blot. (E) CNE2/DDP cells were untreated or treated with nedaplatin at indicated concentrations in the absence or presence of Baf A1 (10 nM) for 48h. The cell viability was determined by MTT assay at the wavelength of 570 nm (n = 5, means±SD, **p<0.01, ***p<0.001 vs. each respective nedaplatin group).
(TIF)

S4 Fig The effect of ERK on Akt/mTOR and ROS in HNE1/DDP cells treated with nedaplatin. (A) HNE1/DDP cells were treated with 6.0 μg/ml nedaplatin for 48 h with or without the pretreatment of U0126 (20 μM) for 2 h. Levels of pAkt, pmTOR were detected by western blot. (B) HNE1/DDP cells were incubated with 6.0 μg/ml nedaplatin in the presence or absence of U0126 (20 μM) for 12 h. Then, the samples were prepared as described in the “Materials and methods” section. All data are expressed as means ± SD of five independent experiments.
(TIF)

S1 Original Original for PLOS ONE. (ZIP)
