# Supplementary figures and images for: Inhibition of Autophagy Potentiated the Antitumor Effect of Nedaplatin in Cisplatin-Resistant Nasopharyngeal Carcinoma Cells
Source: PLoS One. 2015 Aug 19;10(8):e0135236. doi: 10.1371/journal.pone.0135236 (PMC4543554; doi:10.1371/journal.pone.0135236)

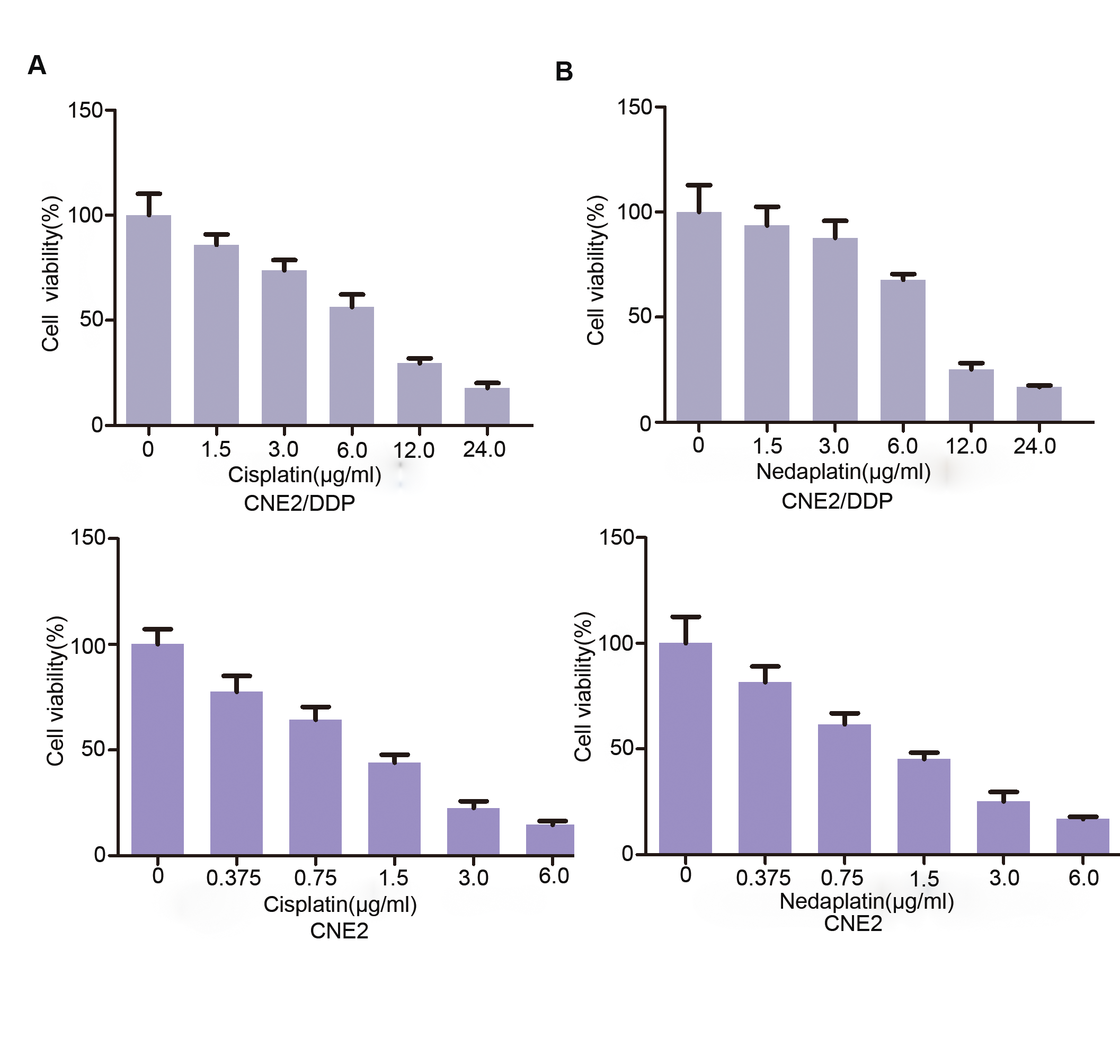

Supplement: S1 Fig — (A) CNE2 cells and CNE2/DDP cells were treated with the indicated concentrations of cisplatin for 48 h. The cell viability was determined by MTT assay at the wavelength of 570 nm. (B) CNE2 cells and CNE2/DDP cells were treated with the indicated concentrations of nedaplatin for 48 h. The cell viability was determined by MTT assay at the wavelength of 570 nm. (TIF) [file pone.0135236.s001.tif]

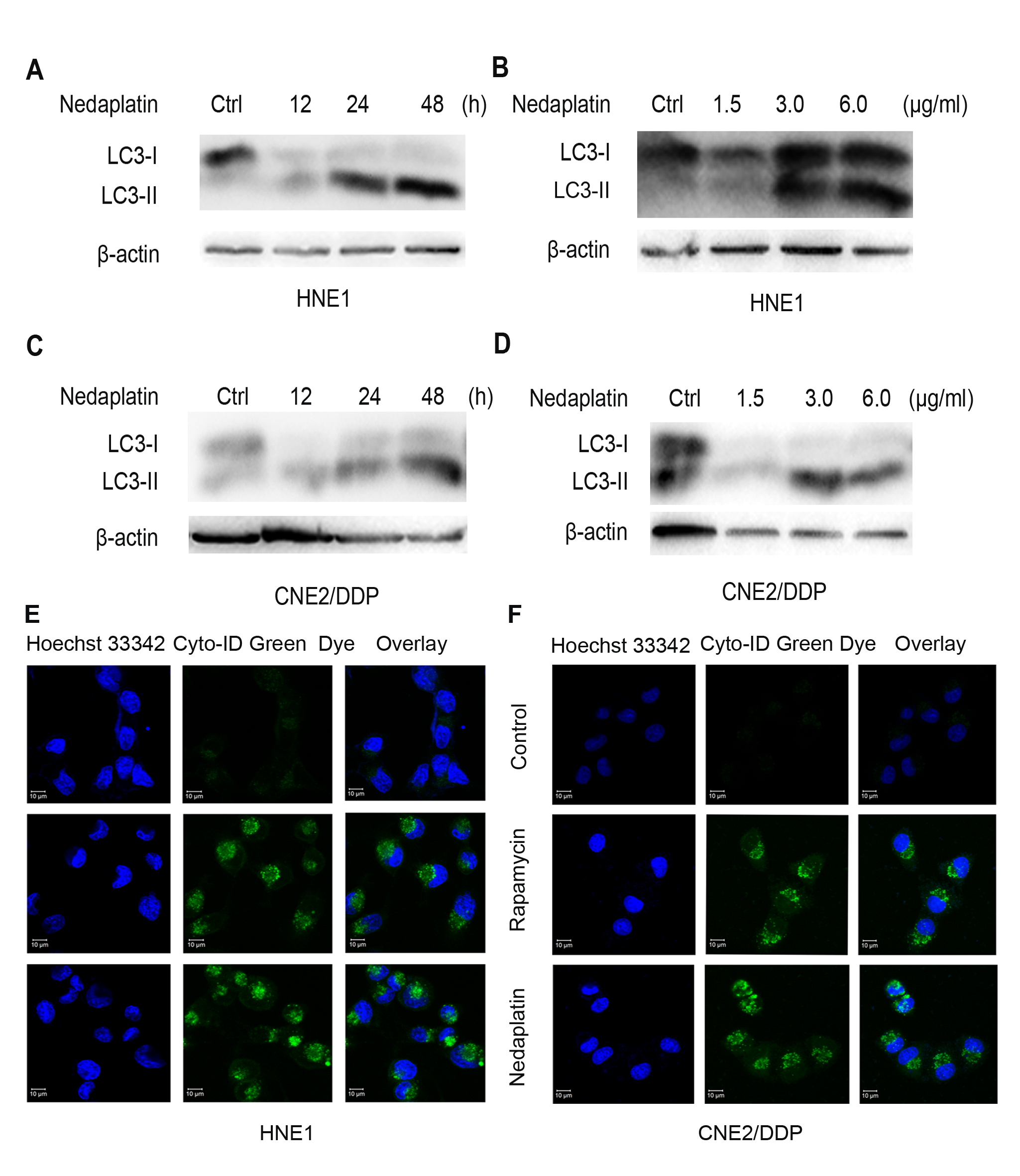

Supplement: S2 Fig — (A) Immunoblot analysis of LC3-I/II levels. HNE1 cells were treated with 6.0 μg/ml nedaplatin for 12, 24, and 48 h. (B) Immunoblot analysis of LC3-I/II levels. HNE1 cells were treated with nedaplatin for 48 h at 0, 1.5, 3.0 and 6.0 μg/ml. (C) Immunoblot analysis of LC3-I/II levels. CNE2/DDP cells were treated with 6.0 μg/ml nedaplatin for 12, 24, and 48 h. (D) Immunoblot analysis of LC3-I/II levels. CNE2/DDP cells were treated with nedaplatin for 48 h at 0, 1.5, 3.0 and 6.0 μg/ml.(E) HNE1 cells were treated with 6.0 μg /ml of nedaplatin for 24 h or with 500 nM of rapamycin for 4 h, and then the cells were stained with Cyto-ID Green autophagy dye and analyzed by confocal microscopy. (F) CNE2/DDP cells were treated with 6.0 μg /ml of nedaplatin for 24 h or with 500 nM of rapamycin for 4 h, and then the cells were stained with Cyto-ID Green autophagy dye and analyzed by confocal microscopy. (TIF) [file pone.0135236.s002.tif]

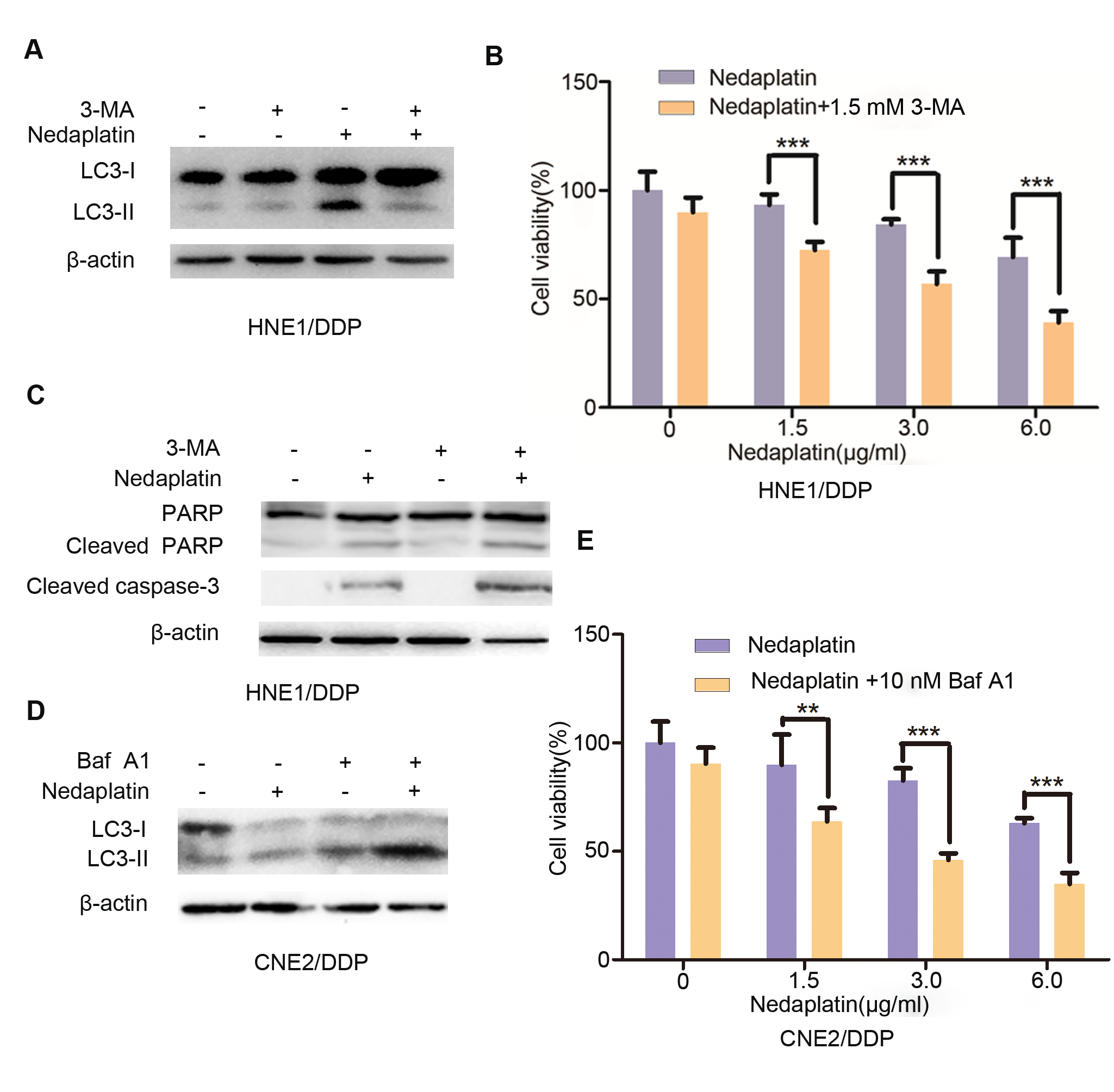

Supplement: S3 Fig — (A) HNE1/DDP cells were incubated with 6.0 μg/ml nedaplatin for 48 h, in the presence or absence of 3-MA (1.5 mM) for 48 h, and the levels of LC3-I/II were detected by western blot. (B) HNE1/DDP cells were untreated or treated with nedaplatin at indicated concentrations in the absence or presence of 3-MA (1.5 mM) for 48h. The cell viability was determined by MTT assay at the wavelength of 570 nm (n = 5, means±SD, ***p<0.001 vs. each respective nedaplatin group). (C) HNE1/DDP cells were incubated with or without 6.0 μg/ml of nedaplatin in the presence or absence of the autophagy inhibitors 3-MA (1.5 mM) for 48 h. The whole protein was extracted, and PARP, cleaved PARP and cleaved caspase-3 were analyzed by western blot. (D) CNE2/DDP cells were incubated with 6.0 μg/ml nedaplatin for 48 h, in the presence or absence of Baf A1 (10 nM) for 48 h, and the levels of LC3-I/II were detected by western blot. (E) CNE2/DDP cells were untreated or treated with nedaplatin at indicated concentrations in the absence or presence of Baf A1 (10 nM) for 48h. The cell viability was determined by MTT assay at the wavelength of 570 nm (n = 5, means±SD, **p<0.01, ***p<0.001 vs. each respective nedaplatin group). (TIF) [file pone.0135236.s003.tif]

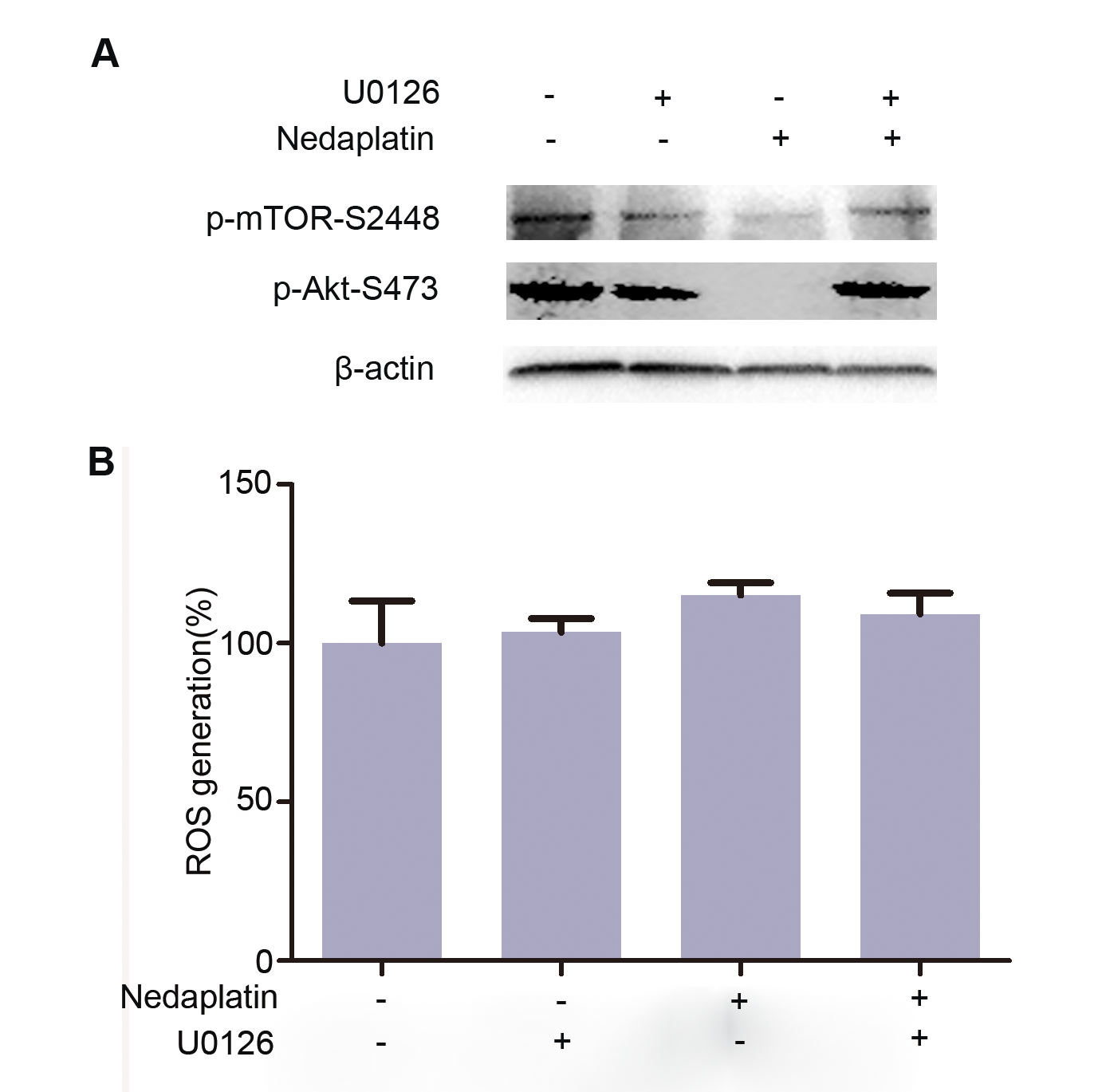

Supplement: S4 Fig — (A) HNE1/DDP cells were treated with 6.0 μg/ml nedaplatin for 48 h with or without the pretreatment of U0126 (20 μM) for 2 h. Levels of pAkt, pmTOR were detected by western blot. (B) HNE1/DDP cells were incubated with 6.0 μg/ml nedaplatin in the presence or absence of U0126 (20 μM) for 12 h. Then, the samples were prepared as described in the “Materials and methods” section. All data are expressed as means ± SD of five independent experiments. (TIF) [file pone.0135236.s004.tif]

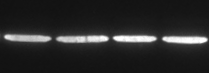

Supplement: S1 Original — (ZIP) [file pone.0135236.s005.zip › S1 Orinigal/Fig 1/Fig 1C HNE1DDP/actin.tif]

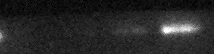

Supplement: S1 Original — (ZIP) [file pone.0135236.s005.zip › S1 Orinigal/Fig 1/Fig 1C HNE1DDP/cleaved caspase-3.tif]

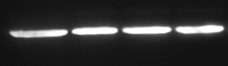

Supplement: S1 Original — (ZIP) [file pone.0135236.s005.zip › S1 Orinigal/Fig 1/Fig 1C HNE1/actin.tif]

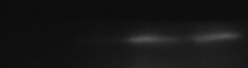

Supplement: S1 Original — (ZIP) [file pone.0135236.s005.zip › S1 Orinigal/Fig 1/Fig 1C HNE1/cleaved caspase-3.tif]

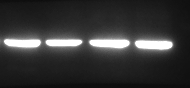

Supplement: S1 Original — (ZIP) [file pone.0135236.s005.zip › S1 Orinigal/Fig 1/Fig 1D HNE1DDP/actin.tif]

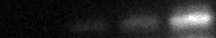

Supplement: S1 Original — (ZIP) [file pone.0135236.s005.zip › S1 Orinigal/Fig 1/Fig 1D HNE1DDP/cleaved casepase-3.tif]

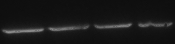

Supplement: S1 Original — (ZIP) [file pone.0135236.s005.zip › S1 Orinigal/Fig 1/Fig 1D HNE1/actin.tif]

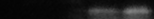

Supplement: S1 Original — (ZIP) [file pone.0135236.s005.zip › S1 Orinigal/Fig 1/Fig 1D HNE1/cleaved caspase-3.tif]

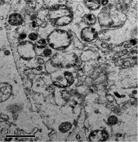

Supplement: S1 Original — (ZIP) [file pone.0135236.s005.zip › S1 Orinigal/Fig 2/Fig 2A/Magnified view.tif]

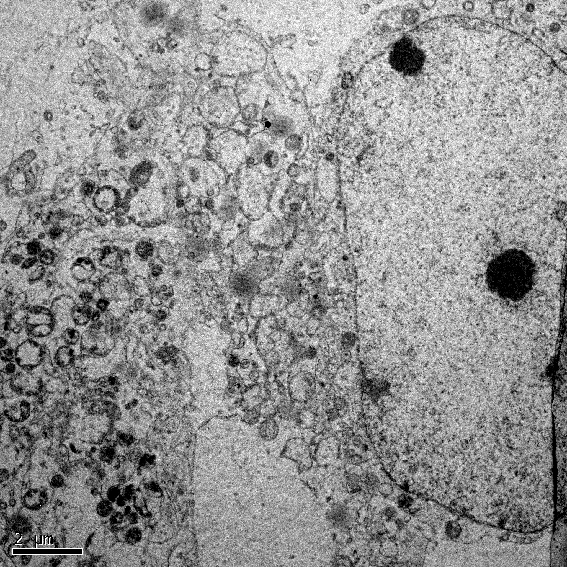

Supplement: S1 Original — (ZIP) [file pone.0135236.s005.zip › S1 Orinigal/Fig 2/Fig 2A/Nedaplatin.tif]

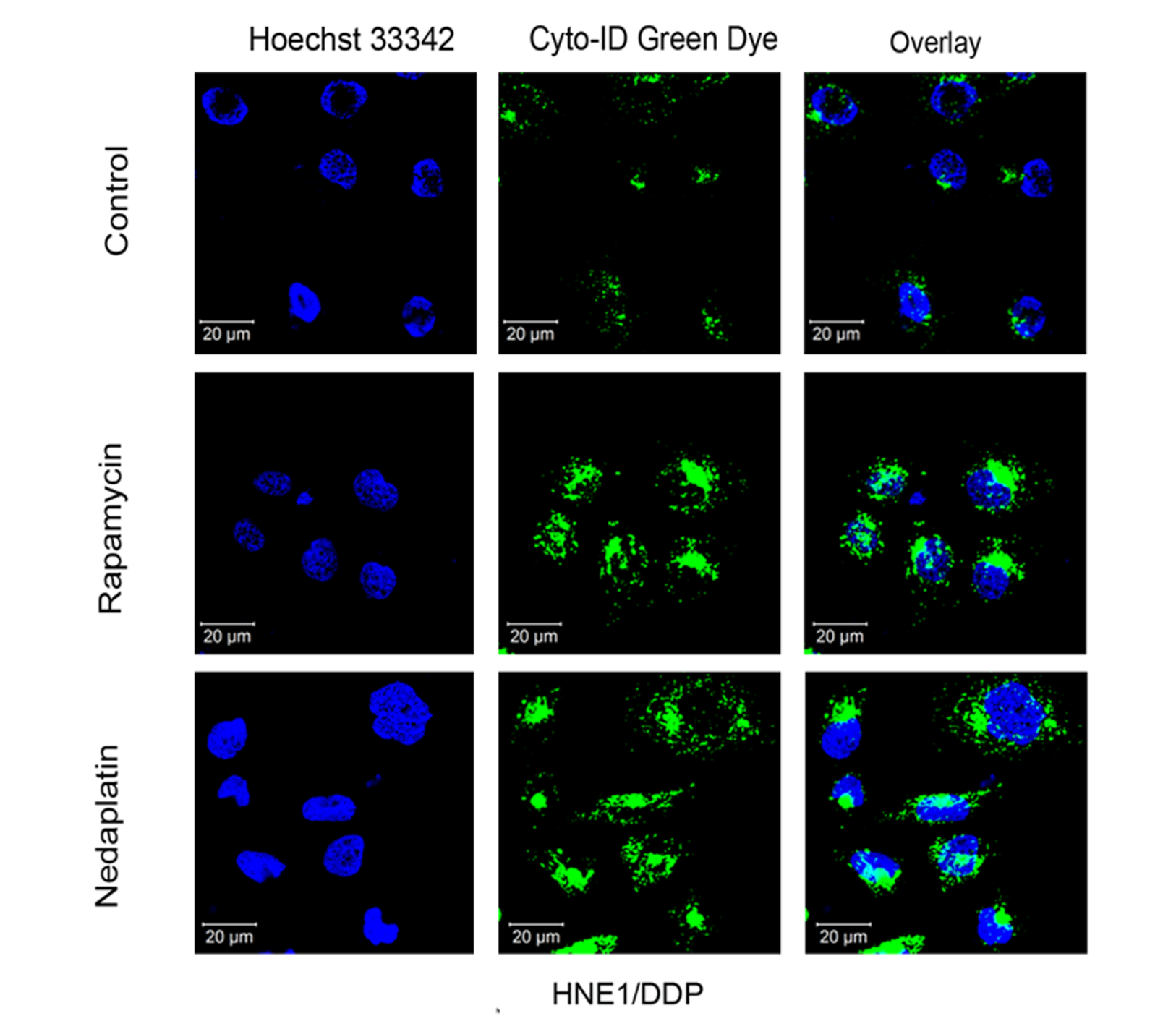

Supplement: S1 Original — (ZIP) [file pone.0135236.s005.zip › S1 Orinigal/Fig 2/Fig 2B.tif]

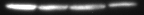

Supplement: S1 Original — (ZIP) [file pone.0135236.s005.zip › S1 Orinigal/Fig 2/Fig 2C/actin.tif]

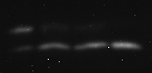

Supplement: S1 Original — (ZIP) [file pone.0135236.s005.zip › S1 Orinigal/Fig 2/Fig 2C/LC3.tif]

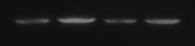

Supplement: S1 Original — (ZIP) [file pone.0135236.s005.zip › S1 Orinigal/Fig 2/Fig 2D/actin.tif]

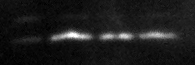

Supplement: S1 Original — (ZIP) [file pone.0135236.s005.zip › S1 Orinigal/Fig 2/Fig 2D/LC3.tif]

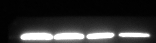

Supplement: S1 Original — (ZIP) [file pone.0135236.s005.zip › S1 Orinigal/Fig 3/Fig 3A/actin.tif]

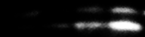

Supplement: S1 Original — (ZIP) [file pone.0135236.s005.zip › S1 Orinigal/Fig 3/Fig 3A/LC3.png]

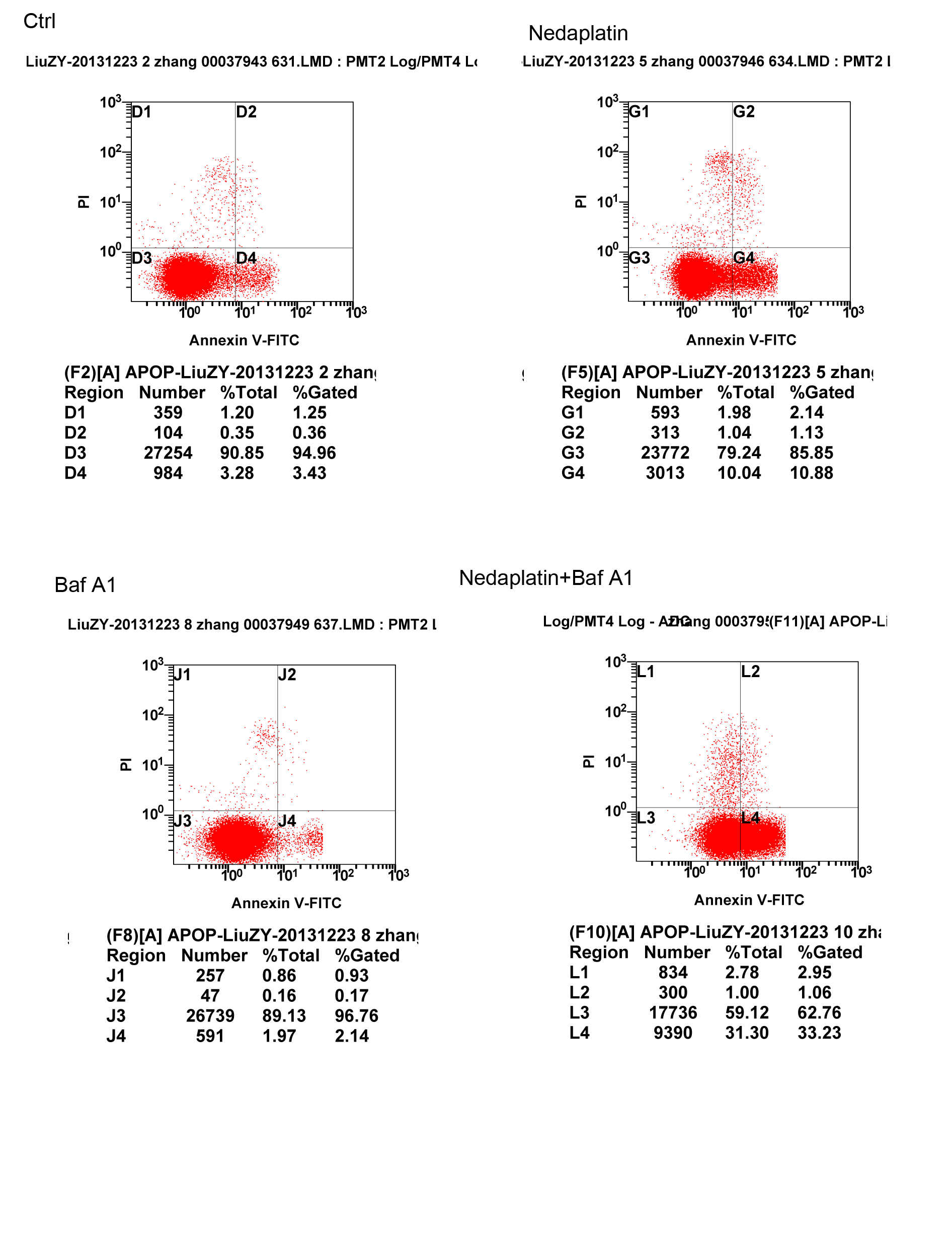

Supplement: S1 Original — (ZIP) [file pone.0135236.s005.zip › S1 Orinigal/Fig 3/Fig 3C.tif]

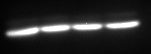

Supplement: S1 Original — (ZIP) [file pone.0135236.s005.zip › S1 Orinigal/Fig 3/Fig 3D/actin.tif]

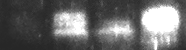

Supplement: S1 Original — (ZIP) [file pone.0135236.s005.zip › S1 Orinigal/Fig 3/Fig 3D/cleaved caepase-3.tif]

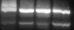

Supplement: S1 Original — (ZIP) [file pone.0135236.s005.zip › S1 Orinigal/Fig 3/Fig 3D/PARP.tif]

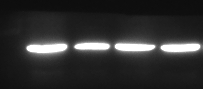

Supplement: S1 Original — (ZIP) [file pone.0135236.s005.zip › S1 Orinigal/Fig 4/Fig 4D/actin.tif]

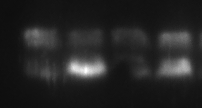

Supplement: S1 Original — (ZIP) [file pone.0135236.s005.zip › S1 Orinigal/Fig 4/Fig 4D/LC3.tif]

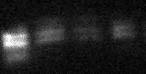

Supplement: S1 Original — (ZIP) [file pone.0135236.s005.zip › S1 Orinigal/Fig 5/Fig 5A/4-EBP1.tif]

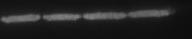

Supplement: S1 Original — (ZIP) [file pone.0135236.s005.zip › S1 Orinigal/Fig 5/Fig 5A/actin.tif]

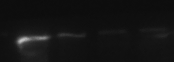

Supplement: S1 Original — (ZIP) [file pone.0135236.s005.zip › S1 Orinigal/Fig 5/Fig 5A/P-70.tif]

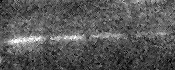

Supplement: S1 Original — (ZIP) [file pone.0135236.s005.zip › S1 Orinigal/Fig 5/Fig 5A/p-Akt.tif]

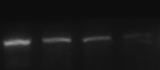

Supplement: S1 Original — (ZIP) [file pone.0135236.s005.zip › S1 Orinigal/Fig 5/Fig 5A/P-mTOR.tif]

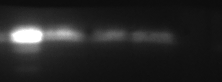

Supplement: S1 Original — (ZIP) [file pone.0135236.s005.zip › S1 Orinigal/Fig 5/Fig 5B/4EBP1.tif]

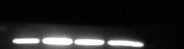

Supplement: S1 Original — (ZIP) [file pone.0135236.s005.zip › S1 Orinigal/Fig 5/Fig 5B/actin.tif]

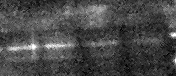

Supplement: S1 Original — (ZIP) [file pone.0135236.s005.zip › S1 Orinigal/Fig 5/Fig 5B/p-Akt.tif]

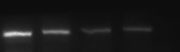

Supplement: S1 Original — (ZIP) [file pone.0135236.s005.zip › S1 Orinigal/Fig 5/Fig 5B/p-mTOR.tif]

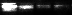

Supplement: S1 Original — (ZIP) [file pone.0135236.s005.zip › S1 Orinigal/Fig 5/Fig 5B/P70.png]

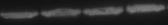

Supplement: S1 Original — (ZIP) [file pone.0135236.s005.zip › S1 Orinigal/Fig 5/Fig 5C/actin.tif]

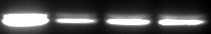

Supplement: S1 Original — (ZIP) [file pone.0135236.s005.zip › S1 Orinigal/Fig 5/Fig 5C/ERK.png]

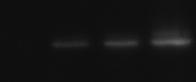

Supplement: S1 Original — (ZIP) [file pone.0135236.s005.zip › S1 Orinigal/Fig 5/Fig 5C/p-Erk.tif]

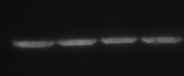

Supplement: S1 Original — (ZIP) [file pone.0135236.s005.zip › S1 Orinigal/Fig 5/Fig 5D/actin.tif]

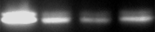

Supplement: S1 Original — (ZIP) [file pone.0135236.s005.zip › S1 Orinigal/Fig 5/Fig 5D/ERK.png]

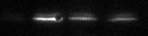

Supplement: S1 Original — (ZIP) [file pone.0135236.s005.zip › S1 Orinigal/Fig 5/Fig 5D/P-ERK.tif]

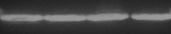

Supplement: S1 Original — (ZIP) [file pone.0135236.s005.zip › S1 Orinigal/Fig 5/Fig 5E/actin.png]

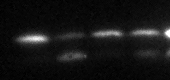

Supplement: S1 Original — (ZIP) [file pone.0135236.s005.zip › S1 Orinigal/Fig 5/Fig 5E/LC3.tif]

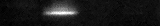

Supplement: S1 Original — (ZIP) [file pone.0135236.s005.zip › S1 Orinigal/Fig 5/Fig 5E/p-Erk.tif]

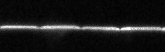

Supplement: S1 Original — (ZIP) [file pone.0135236.s005.zip › S1 Orinigal/Fig 5/Fig 5F/actin.tif]

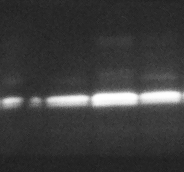

Supplement: S1 Original — (ZIP) [file pone.0135236.s005.zip › S1 Orinigal/Fig 5/Fig 5F/p-Erk.tif]

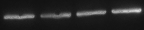

Supplement: S1 Original — (ZIP) [file pone.0135236.s005.zip › S1 Orinigal/S2_Fig/S2A_Fig/actin.tif]

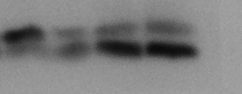

Supplement: S1 Original — (ZIP) [file pone.0135236.s005.zip › S1 Orinigal/S2_Fig/S2A_Fig/LC3.tif]

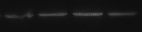

Supplement: S1 Original — (ZIP) [file pone.0135236.s005.zip › S1 Orinigal/S2_Fig/S2B_Fig/actin.tif]

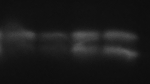

Supplement: S1 Original — (ZIP) [file pone.0135236.s005.zip › S1 Orinigal/S2_Fig/S2B_Fig/LC3.tif]

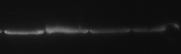

Supplement: S1 Original — (ZIP) [file pone.0135236.s005.zip › S1 Orinigal/S2_Fig/S2C_Fig/actin.tif]

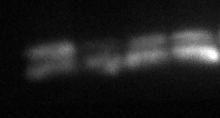

Supplement: S1 Original — (ZIP) [file pone.0135236.s005.zip › S1 Orinigal/S2_Fig/S2C_Fig/LC3.tif]

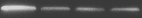

Supplement: S1 Original — (ZIP) [file pone.0135236.s005.zip › S1 Orinigal/S2_Fig/S2D_Fig/actin.tif]

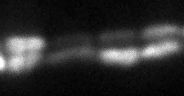

Supplement: S1 Original — (ZIP) [file pone.0135236.s005.zip › S1 Orinigal/S2_Fig/S2D_Fig/LC3.tif]

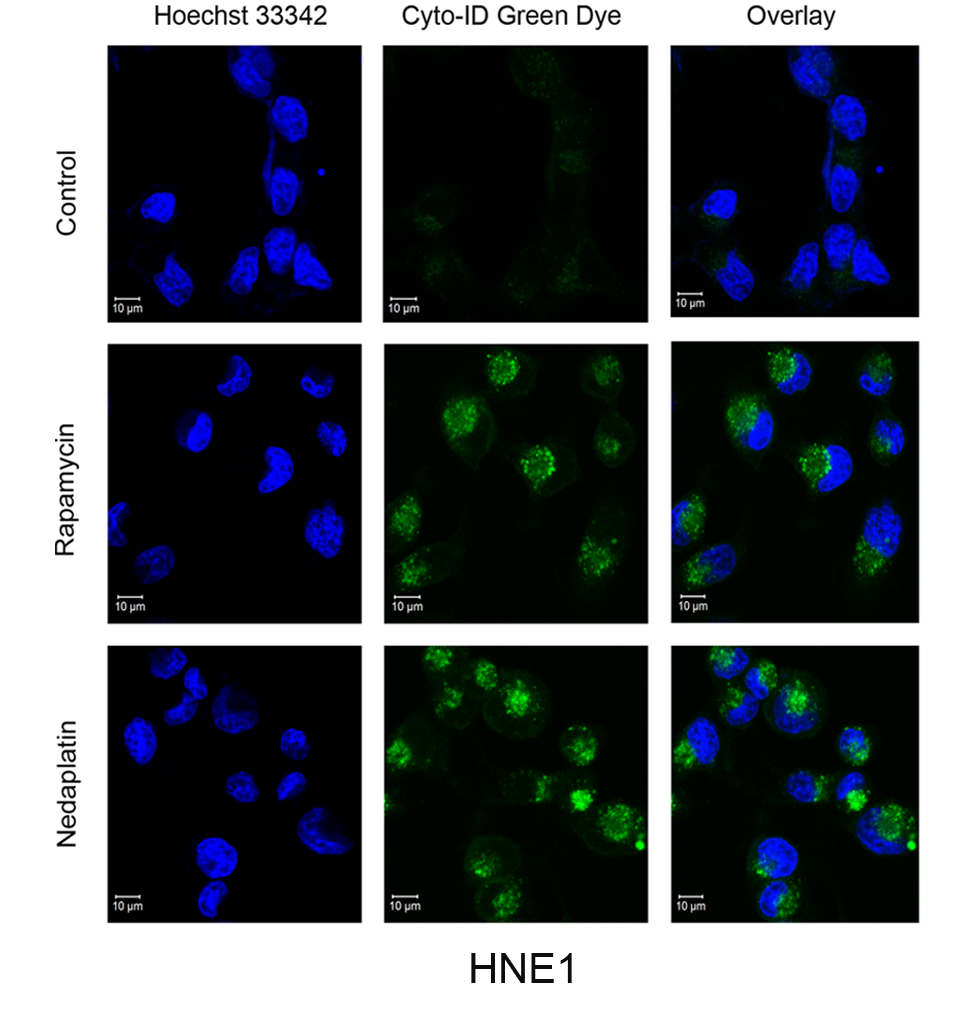

Supplement: S1 Original — (ZIP) [file pone.0135236.s005.zip › S1 Orinigal/S2_Fig/S2E_Fig.tif]

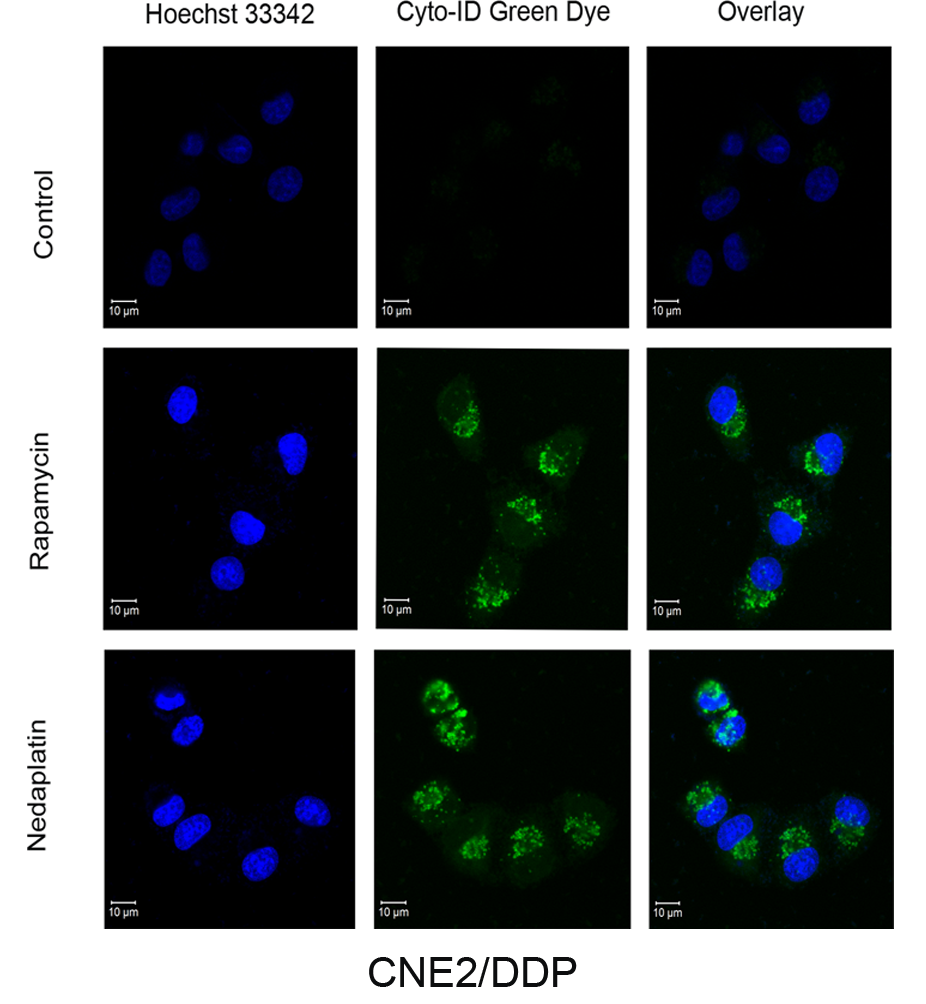

Supplement: S1 Original — (ZIP) [file pone.0135236.s005.zip › S1 Orinigal/S2_Fig/S2F_Fig.tif]

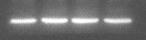

Supplement: S1 Original — (ZIP) [file pone.0135236.s005.zip › S1 Orinigal/S3_Fig/S3A_Fig/actin.tif]

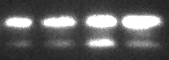

Supplement: S1 Original — (ZIP) [file pone.0135236.s005.zip › S1 Orinigal/S3_Fig/S3A_Fig/LC3.tif]

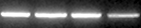

Supplement: S1 Original — (ZIP) [file pone.0135236.s005.zip › S1 Orinigal/S3_Fig/S3C_Fig/actin.tif]

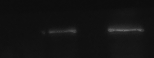

Supplement: S1 Original — (ZIP) [file pone.0135236.s005.zip › S1 Orinigal/S3_Fig/S3C_Fig/cleaved caspase-3.tif]

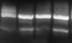

Supplement: S1 Original — (ZIP) [file pone.0135236.s005.zip › S1 Orinigal/S3_Fig/S3C_Fig/parp.tif]

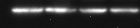

Supplement: S1 Original — (ZIP) [file pone.0135236.s005.zip › S1 Orinigal/S3_Fig/S3D_Fig/actin.tif]

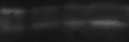

Supplement: S1 Original — (ZIP) [file pone.0135236.s005.zip › S1 Orinigal/S3_Fig/S3D_Fig/LC3.tif]

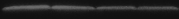

Supplement: S1 Original — (ZIP) [file pone.0135236.s005.zip › S1 Orinigal/S4_Fig/S4A_Fig/actin.tif]

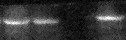

Supplement: S1 Original — (ZIP) [file pone.0135236.s005.zip › S1 Orinigal/S4_Fig/S4A_Fig/p-Akt.tif]

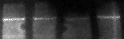

Supplement: S1 Original — (ZIP) [file pone.0135236.s005.zip › S1 Orinigal/S4_Fig/S4A_Fig/p-mTOR.tif]
